# A novel platform for isotype-specific testing of autoantibodies

**DOI:** 10.1371/journal.pone.0211596

**Published:** 2019-02-07

**Authors:** Kaylene L. Carter, Anckia Treurnicht, Kara L. Davis, Rajiv B. Kumar, Brian J. Feldman

**Affiliations:** 1 Department of Pediatrics, Stanford School of Medicine, Stanford, California, United States of America; 2 Stanford Cardiovascular Institute, Stanford School of Medicine, Stanford, California, United States of America; 3 Department of Pediatrics, University of California, San Francisco, San Francisco, California, United States of America; University of Miami, UNITED STATES

## Abstract

The objective of this study was to test if a novel platform could be used for isotype-specific autoantibody testing in humans. Further, we evaluated if testing with this novel platform enables earlier detection of insulin autoantibodies in individuals that have first-degree relatives with type-1 diabetes than currently used approaches. Longitudinal serum samples from participants were collected before and after they converted to become positive for insulin autoantibodies by the current standardly used assays. Using a novel plasmonic gold chip platform, we tested these samples for IgM isotype-specific autoantibodies. Serial serum samples from individuals without diabetes were also tested as a comparison control cohort. Our results demonstrate proof-of-concept that a plasmonic gold chip can specifically detect the IgM insulin autoantibody. Five out of the six individuals that converted to being positive for insulin autoantibodies by standard testing had significant IgM autoantibodies on the plasmonic chip platform. The plasmonic chip platform detected IgM autoantibodies earlier than standard testing by up to 4 years. Our results indicate that the plasmonic gold platform can specifically detect the IgM isotype autoantibodies and suggest that combining isotype-specific testing with currently used approaches enables earlier detection of insulin autoantibodies in individuals that have first-degree relatives with type 1 diabetes.

## Introduction

Autoimmune diseases are a diverse collection of disorders of the immune system where self-antigens induce a pathological response in the body including the production of autoantibodies (AAbs) [1]. The resulting symptoms of this process are dependent on a variety of factors, including which antigens are triggering the response, and span the spectrum of severity from causing premature greying of hair to life threatening systemic disease [2]. In most autoimmune diseases, rapid diagnosis is critical for clarifying the cause of symptoms as well as initiating appropriate therapy. Detection of AAbs to particular antigens is often essential for making a diagnosis of an autoimmune disease. In some cases, continued testing for AAbs is helpful to monitor the response to therapy.

With few exceptions, the most widely used method for detecting AAbs has been the use the enzyme-linked immunosorbent assay (ELISA) platform. However, unless specifically adapted, standard ELISA assays are either biased or exclusively dedicated to the detection of the IgG isotype of the AAbs. In many cases of long standing autoimmune disease in a patient, this does not impair the ability to make the diagnosis and therefore has been broadly adapted and useful. However, classically, activated B-cells will initially secrete IgM isotype antibodies prior to class-switching to IgG [3]. Therefore, there is a potential window where platforms biased toward IgG detection may miss early stage autoimmune disease. As clinical knowledge advances and therapies develop, it is a common objective to diagnose and initiate therapy as rapidly as possible to decrease morbidity and increase the efficacy of therapies. In this regard, increased testing for IgM AAbs may enable earlier detection of disease and lead to better patient outcomes. In addition, there are particular situations where the detection of specific AAbs can be used as biomarkers that predict the development of disease in the future. These scenarios provide an opportunity to intervene prior to the onset of symptoms. In these situations, where disease is early stage, and potentially even pre-symptomatic, we are concerned that assays that are designed to detect IgG isotypes might be prone to failing to identify at-risk individuals. We hypothesize that using platforms that are competent to detect IgM isotypes of AAbs would be particularly useful for identifying individuals in this early phase of the natural history of the disease.

Type-1 diabetes (T1D) is a compelling disease to begin to test these hypotheses. T1D results from autoimmune destruction of insulin secreting islet cells in the pancreas leading to insulin deficiency and the inability of the body to effectively import glucose from the circulation into tissues. There is currently no cure for T1D and treatment requires lifelong daily administration of exogenous insulin guided by frequent monitoring of blood sugar concentrations. The detection of AAbs to specific islet antigens is a critical element of establishing the diagnosis of T1D. For unclear reasons, the incidence of T1D has been increasing in both children and adults [4–6]. To address this important and growing need, there are significant efforts underway to develop better therapies and ultimately a cure for T1D. A variety of types and combinations of next-generation therapies under development are showing increasing promise toward modifying the pathological immune processes and preventing disease progression. A common finding of these studies is that the sooner therapies are initiated from the time of diagnosis, the higher the efficacy [7–9]. Initiating therapy as early as possible is also consistent with the longer-term goal of intercepting the pathogenesis of T1D prior to when symptoms develop.

The feasibility of identifying individuals that will go on to develop T1D was established by the robust predictive value of detecting AAbs against islet antigens [10,11]. With this strong correlation, leading groups in the T1D scientific communities issued a consensus statement that supports moving the detection of islet AAbs from being classified as a biomarker for the risk of future disease to becoming diagnostic for what is termed ‘pre-symptomatic diabetes’ to reflect the progression that the presence of islet AAbs foretell [12].

Together, these advances continue to elevate the importance of monitoring islet AAbs and identifying the earliest biomarkers for T1D risk and disease progression. Despite all of these compelling reasons to further investigate IgM isotypes in patients with, or at-risk for T1D, the detection of islet AAbs, particularly insulin AAbs, using standard platforms such as ELISA has not been successful. As a results of the failure of ELISA in this situation, the current gold-standard platform that is used across clinical and many research laboratories to detect islet AAbs is the radioimmunoassay (RIA) that use Protein A Sepharose [13], which is biased toward measuring the IgG isotypes of the AAbs. This has created a technological obstacle to conducting important studies on the IgM isotype in patients with or at-risk for developing T1D.

We hypothesized that a novel platform we previously developed [14], that is competent to detect islet AAbs, could be adapted to specifically measure the IgM isotype of insulin AAbs. If successful, this technological advance would enable broad studies that could determine if IgM isotypes of islet AAbs were developing in patients prior to the onset of symptoms that were eluding detection by RIA.

## Materials and methods

These studies were approved by the Stanford Institutional Review Board as well as by the TrialNet Ancillary Study Committee. Informed signed consent was obtained from participants.

Isotype-specific insulin AAbs in the plasma samples were detected on a plasmonic chip as previously described [14]. Briefly, de-identified samples were received from the TrialNet Biobank blinded (no information about when the volunteers became RIA positive was sent with the samples) and then aliquoted, to maintain the same number of freeze-thaw cycles between replicates, and stored at -80°C. Samples from controls were similarly aliquoted and stored at -80°C. On the day of testing, an aliquot from each of the time-points from a donor was thawed and used to probe a plasmonic chip, which was arrayed with antigens in triplicate. The chips were then washed and probed with secondary Cy3 conjugated anti-IgM antibodies (Jackson ImmunoResearch) followed by a final washing. The full time-course from each donor was tested on the same chip. The mean fluorescence intensity (MFI) of the triplicate arrays was calculated for each time-point by scanning the arrays using a GenePix 4000B scanner and GenePix Pro 6.1 software. Testing of the entire time-course for each donor was replicated 3 or 4 times, each time on a separate day and on a plasmonic chip generated from a distinct production run. The results of our blinded testing of the samples were sent to TrialNet prior to unblinding the results of their testing of the samples by RIA.

### Statistical analysis

The mean of the signals from the triplicate arrayed antigens was used to determine the MFI for each time-point. The entire time-course for each donor was repeated either 3 or 4 times and the means of the MFIs for the replicates were calculated for each time-point. Paired t-tests were used to compare the time-point to the intra-individual nadir MFI for the time-course to test for significant changes in IgM levels. IgM positivity was defined by having an MFI value at least 2.5-fold greater than that of the intra-course nadir, along with a *p* < 0.05.

## Results

**Fig 1:**
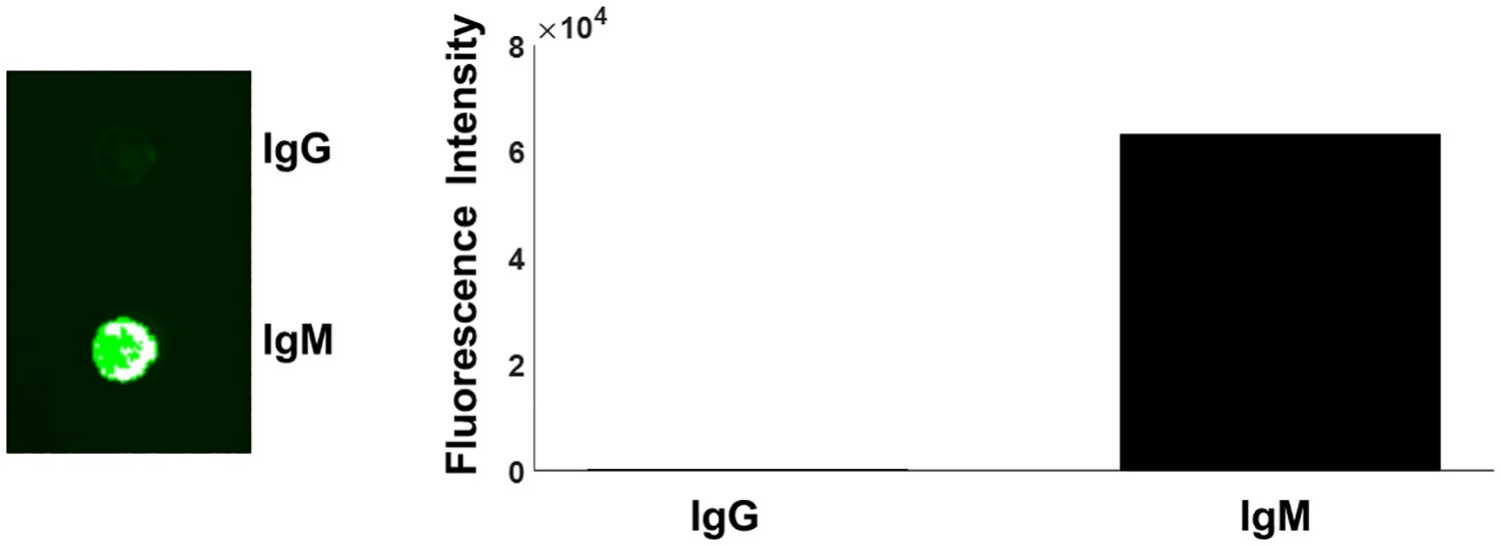
Specific detection of IgM antibodies using the plasmonic chip. (Left) Example image of a plasmonic chip with IgG (upper) and IgM (lower) antibodies arrayed and tested using serum from volunteer followed by probing with Cy3 conjugated anti-IgM antibodies demonstrates specificity for detecting IgM with extremely low cross-reactive detection of IgG isotype antibodies. (Right) Quantification of the fluorescence intensity of the signal from IgG and IgM arrayed spots using a Genepix 4000B scanner.

We previously developed a plasmonic-based platform that is able to detect islet AAbs [14]. We tested if this platform was able to selectively detect isotype specific IgM insulin AAbs for this study. This testing confirmed that the platform could robustly detect the IgM isotype of insulin AAbs with extremely low cross-recognition of the IgG isotype (Fig 1).

Using the TrialNet biobank of longitudinally collected serum samples from asymptomatic insulin naïve first-degree relatives of patients with T1D we obtained serial samples from 6 individuals who entered the TrialNet study negative for any islet AAbs but converted to insulin AAb positive by RIA while participating in the study. We chose to test for insulin AAb because it cannot be reliably tested for using the ELISA platform and is a common islet AAb in diabetes and, therefore, broadly relevant. In addition, because of the high frequency of this AAb, TrialNet was able to provide us with samples from 6 individuals who converted from being islet AAb negative to positive for the insulin AAb during their surveillance. Because of the limited supply of these biobanked samples, we were unable to obtain sufficient numbers of samples from volunteers that went on to become positive to other islet AAbs. For controls, we prospectively collected samples from volunteers without diabetes at Lucile Packard Children’s Hospital at Stanford. We recruited volunteers that were similar ages to the participants that provided the samples we used from the TrialNet biobank (Table 1). In order to survey for changes in IgM levels over time, we also obtained a second timepoint from each of the controls, 2–3 months after their initial sample was collected.

**Table 1:** Volunteer characteristics.

Characteristic	Non-Diabetic Controls (N = 8)	Trial Net Participants (N = 6)
**Age—years (mean)**	6–16.6 (9.4)	3–13.2 (7.2)
**Female sex—no. (%)**	3 (37.5)	4 (66.7)

**Fig 2:**
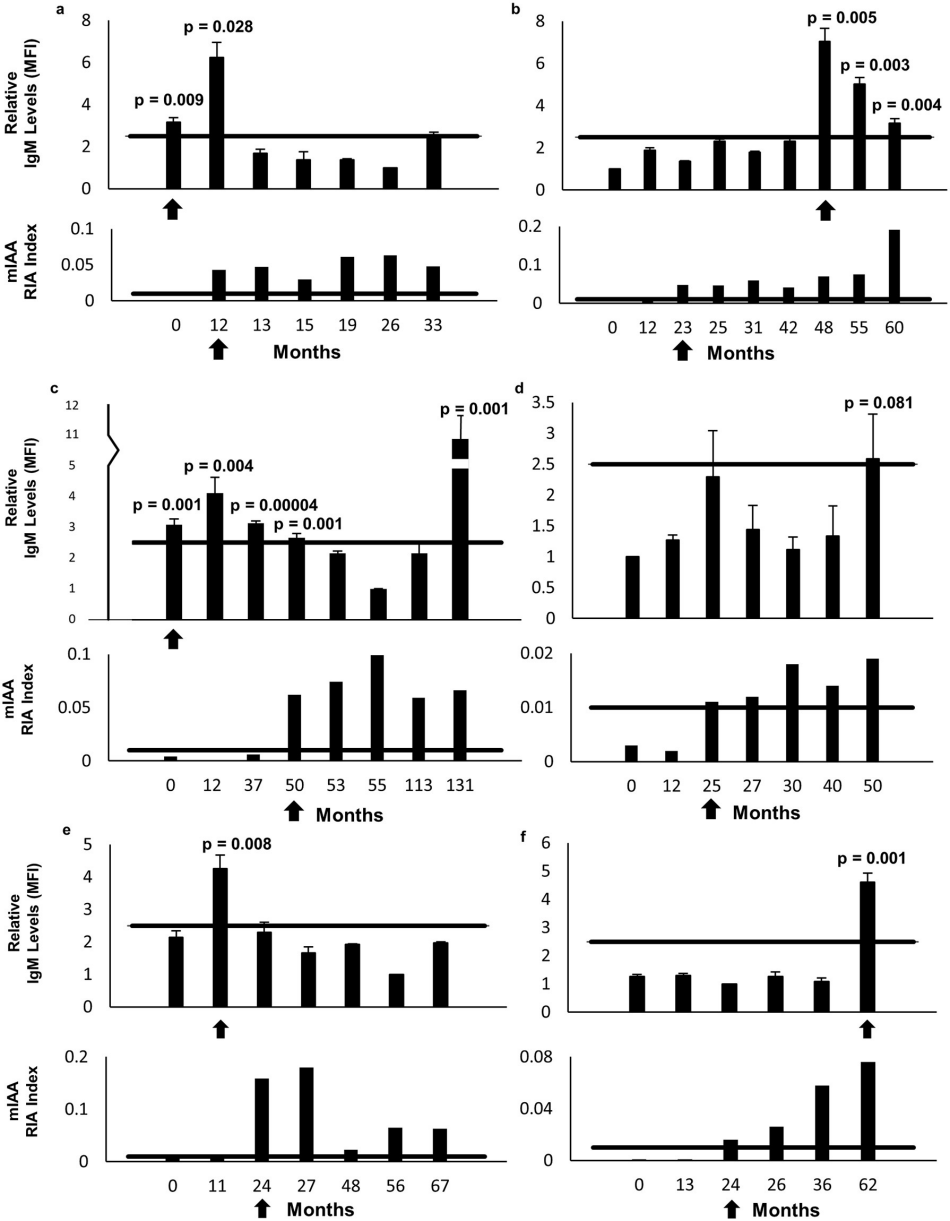
Serial isotype-specific testing for insulin autoantibodies. (**a-f**) Longitudinal IgM isotype-specific test results (upper) from 6 volunteers that became RIA positive (lower). Time was calculated in months from the first available sample. Each test was performed in triplicate to generate the mean fluorescence intensity (MFI) for the time-point. The entire time-course for each individual was repeated 3–4 times in independent experiments and the averages of the MFIs were calculated. MFI values were normalized to each volunteer’s intra-course nadir and expressed as fold-change from the nadir. Error bars represent standard deviations of the replicates, each of which was performed in triplicate. Arrows on upper graphs identify the earliest time-point where IgM levels were > 2.5-fold above nadir (horizontal bar) with a *p* < 0.05. Arrows on lower graphs identify the earliest time-point where RIA was positive (mIAA RIA index > 0.01) by TrialNet testing.

Using the samples from the individuals who converted from islet AAb negative to insulin AAb positive by RIA testing during their surveillance (independently confirmed by testing at TrialNet), 5 of the 6 individuals met the criteria (see Methods) for having a significant increase in their level of IgM insulin AAbs. Three of the 5 individuals became IgM positive earlier than they became RIA positive, by up to 4 years with a mean of 25 months earlier than RIA. Of note, the IgM levels from these individuals had a pattern of progressively elevating to a peak followed by decreasing to a nadir that is consistent with antibody isotype class switching (Fig 2). Importantly, none of the individuals in the control group met criteria for a significant change in their IgM levels (S1 Data).

## Discussion

Our study demonstrates proof-of-concept that it is possible to selectively detect IgM insulin AAbs using a plasmonic gold chip platform. This technological advance enables larger studies on the role of IgM in autoimmune disease in general as well as having specific implication for screening, predicting and diagnosing symptomatic and pre-symptomatic T1D in particular. The platform’s ability to detect isotype specific AAbs in ultra-small blood volumes (less than a drop of blood) as well as the broadly accessible and rapid processing times for this novel approach could enable a variety of previously infeasible studies. We anticipate that studies enabled by this technology will expose the importance and kinetics of isotype specific AAbs in a variety of clinical and research contexts.

In addition, our results suggest that IgM insulin AAbs are generated and can become significant prior to RIA testing. If confirmed in future studies, our findings would represent an important advance for extending how early individuals at-risk for T1D can be identified compared to current capabilities. While 2 of the 6 individuals with TrialNet samples had significant IgM levels detected after RIA positivity it is possible that, in these individuals, an earlier IgM response was missed due to sampling frequency or they may represent a subclass of T1D with a distinct immune response.

Early identification of individuals at-risk for progressing to T1D is likely to decrease morbidity and increase the efficacy of therapies that are currently being tested and improve the feasibility of developing interventions that prevent T1D in the future. Our study supports that isotype-specific monitoring of islet AAbs should be further studied as a means to facilitate earlier detection and improved treatments. Therefore, we propose that this technology could be considered to augment currently used assays to identify individuals as early as possible. Furthermore, our prior studies have demonstrated that it is feasible to perform simultaneous multiplexed testing for both IgM and IgG autoantibodies using a single blood sample [14]. We believe this approach would enable the identification of patients missed by assays that favor IgM or IgG without adding significant cost or time. Therefore, we conclude that this technology enables further exploration of the value of isotype-specific testing in patients with T1D and provokes analogous considerations for other autoimmune diseases, which could also be tested for using the plasmonic chip platform.

## Supporting information

S1 Data Serial isotype-specific testing for insulin autoantibodies in control volunteers. IgM isotype-specific test results from 8 individuals (a-h) without diabetes (controls) during 2 separate serum collections, 2–3 months apart. Each test was performed in triplicate to generate the mean fluorescence intensity (MFI) for the time-point. Both serum samples for each individual were tested 3 times in independent experiments and the averages of the MFIs were calculated.
(PDF)
