# Supplementary figures and images for: A novel platform for isotype-specific testing of autoantibodies
Source: PLoS One. 2019 Feb 7;14(2):e0211596. doi: 10.1371/journal.pone.0211596 (PMC6366878; doi:10.1371/journal.pone.0211596)

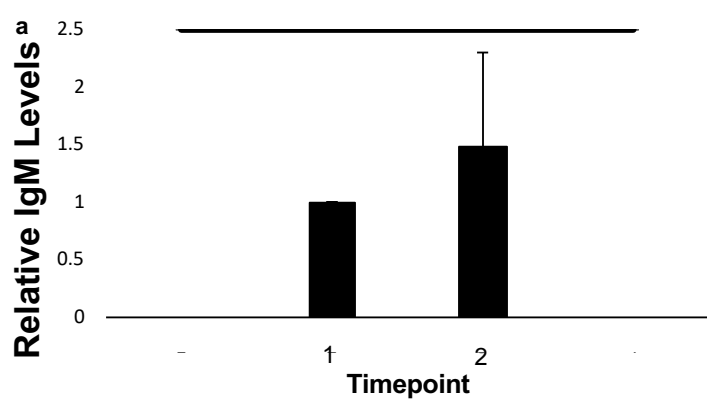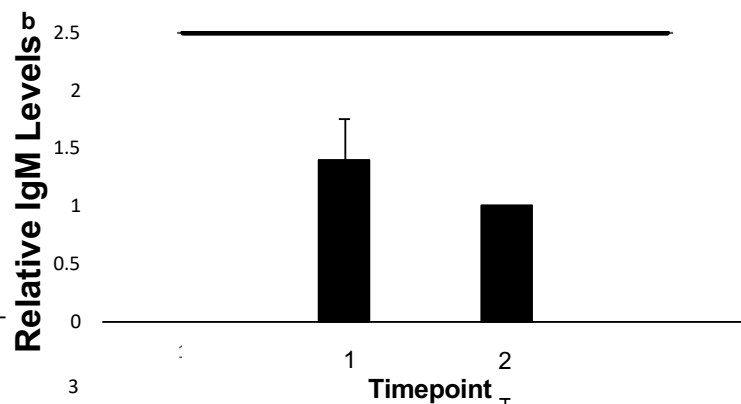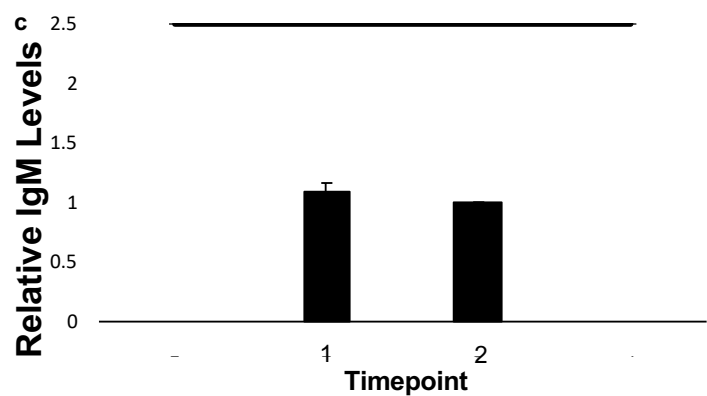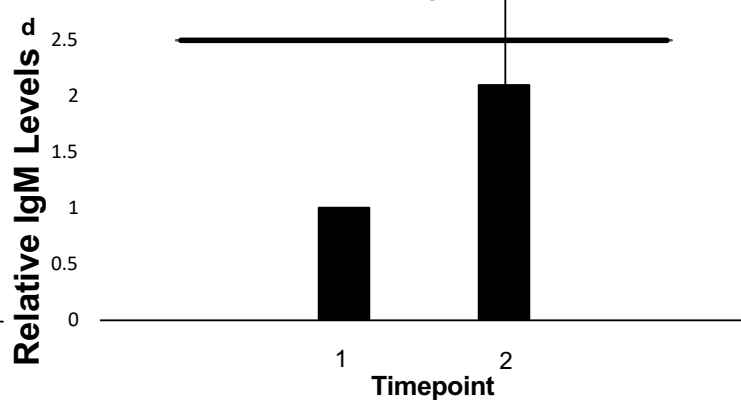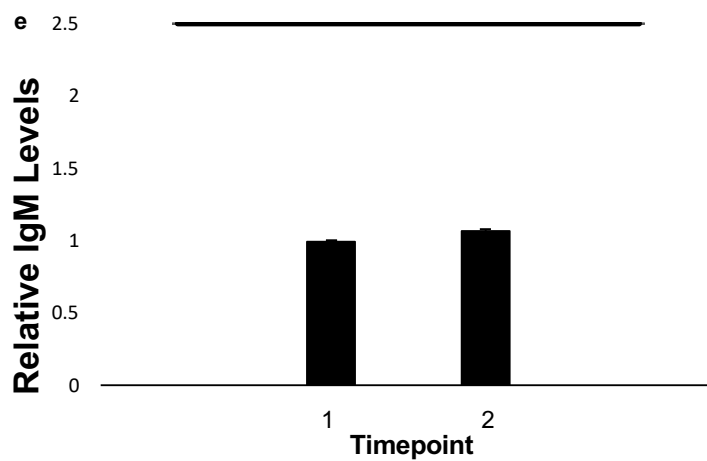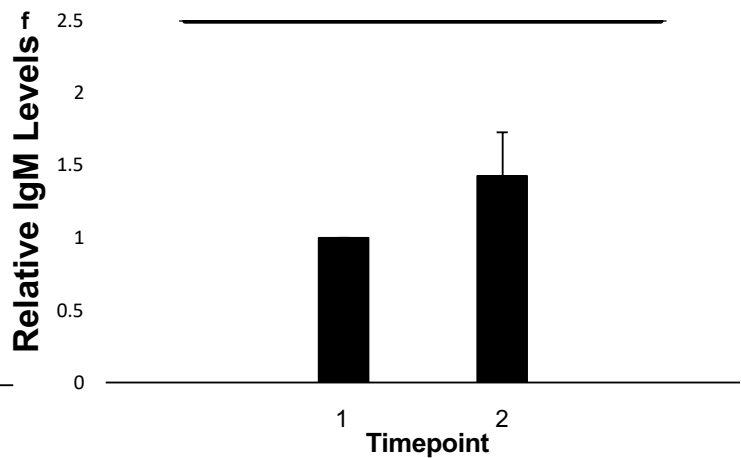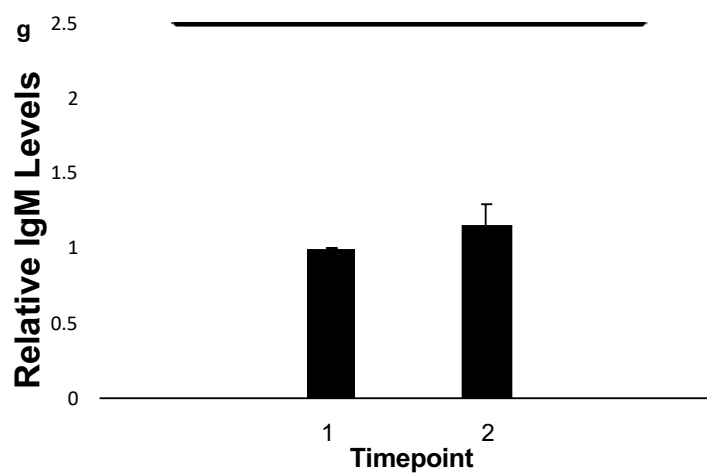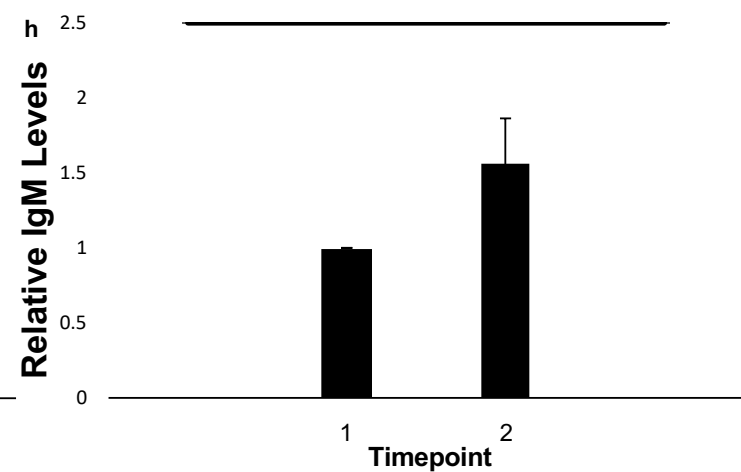

Supplement: S1 Data — IgM isotype-specific test results from 8 individuals (a-h) without diabetes (controls) during 2 separate serum collections, 2–3 months apart. Each test was performed in triplicate to generate the mean fluorescence intensity (MFI) for the time-point. Both serum samples for each individual were tested 3 times in independent experiments and the averages of the MFIs were calculated. (PDF) [file pone.0211596.s001.pdf]
